# Mitochondria-targeted dodecyltriphenylphosphonium (C_12_TPP) combats high-fat-diet-induced obesity in mice

**DOI:** 10.1038/ijo.2016.146

**Published:** 2016-09-20

**Authors:** A V Kalinovich, C L Mattsson, M R Youssef, N Petrovic, M Ost, V P Skulachev, I G Shabalina

**Affiliations:** 1 Department of Molecular Biosciences, The Wenner-Gren Institute, Stockholm University, Stockholm, Sweden; 2 The Belozersky Institute of Physico-Chemical Biology, Lomonosov Moscow State University, Moscow, Russian Federation

## Abstract

**Background:**

A membrane-penetrating cation, dodecyltriphenylphosphonium (C_12_TPP), facilitates the recycling of fatty acids in the artificial lipid membrane and mitochondria. C_12_TPP can dissipate mitochondrial membrane potential and may affect total energy expenditure and body weight in animals and humans.

**Methods:**

We investigated the metabolic effects of C_12_TPP in isolated brown-fat mitochondria, brown adipocyte cultures and mice *in vivo*. Experimental approaches included the measurement of oxygen consumption, carbon dioxide production, western blotting, magnetic resonance imaging and bomb calorimetry.

**Results:**

In mice, C_12_TPP (50 μmol per (day•kg body weight)) in the drinking water significantly reduced body weight (12%, *P*<0.001) and body fat mass (24%, *P*<0.001) during the first 7 days of treatment. C_12_TPP did not affect water palatability and intake or the energy and lipid content in feces. The addition of C_12_TPP to isolated brown-fat mitochondria resulted in increased oxygen consumption. Three hours of pretreatment with C_12_TPP also increased oligomycin-insensitive oxygen consumption in brown adipocyte cultures (*P*<0.01). The effects of C_12_TPP on mitochondria, cells and mice were independent of uncoupling protein 1 (UCP1). However, C_12_TPP treatment increased the mitochondrial protein levels in the brown adipose tissue of both wild-type and UCP1-knockout mice. Pair-feeding revealed that one-third of the body weight loss in C_12_TPP-treated mice was due to reduced food intake. C_12_TPP treatment elevated the resting metabolic rate (RMR) by up to 18% (*P*<0.05) compared with pair-fed animals. C_12_TPP reduced the respiratory exchange ratio, indicating enhanced fatty acid oxidation in mice.

**Conclusions:**

C_12_TPP combats diet-induced obesity by reducing food intake, increasing the RMR and enhancing fatty acid oxidation.

## Introduction

Obesity and its comorbidities represent a global health threat and a rapidly increasing burden to economic prosperity. The development of pharmacologic agents for the treatment of obesity has been challenged by both low efficacy and serious adverse side effects, leading to the removal of these agents from the market.^1^ Despite the checkered past of obesity drug development, the search remains focused on an elusive molecule that might specifically target obesity and effectively prevent its consequences.

Mitochondria occupy a key position in cellular metabolism. The proton motive force across the mitochondrial inner membrane drives ATP synthesis. In addition, the energy stored in the proton motive force can be dissipated by proton leak through the inner membrane, contributing to the basal metabolic rate and thermogenesis.^2, 3, 4^ Protonophores such as 2, 4-dinitrophenol (DNP) increase mitochondrial proton leakage and have been used to treat obesity.^5, 6^ However, a slight increase in the DNP concentration above the therapeutically effective individual dose is toxic and DNP was consequently withdrawn from clinical use.^7^ Thus, considerable interest remains in the development safer protonophores with a wider range of therapeutic concentrations.

Several design concepts for the development of alternative cationic protonophores have been used.^8, 9, 10, 11^ We recently demonstrated that one such cationic protonophore, a short-chain alkyl derivative of rhodamine 19, exhibits metabolic effects in obese mice and consequently may be a promising anti-obesity agent.^12^

Alternatively, mitochondrially targeted uncouplers can be designed by linking the uncoupler moiety to the triphenylphosphonium cation.^9, 10^ MitoDNP, which was designed using this approach, exhibited extensive mitochondrial uptake but did not uncouple mitochondria, as evidenced by its inability to either increase their respiration rate or to decrease membrane potential.^9^ In contrast, another mitochondrially targeted compound conjugated to triphenylphosphonium cation, dodecyltriphenylphosphonium (C_12_TPP), exhibits protonophorous activity in the lipid bilayer membrane and isolated mitochondria.^10^ The uncoupling activity of C_12_TPP was proposed to be mediated by the recycling of endogenous fatty acids, which are natural uncoupling agents in the mitochondrial membrane.^10^ In this work, we studied the potential efficacy of C_12_TPP as an anti-obesity agent.

## Materials and methods

### Animals and treatment

Uncoupling protein 1 (UCP1)-ablated mice that were progeny of mice described in Enerbäck *et al.*^13^ were backcrossed to the C57Bl/6 mouse strain, which was used as the wild-type mouse strain. Male mice were singly housed, had free access to water and were maintained on a 12:12 h light:dark cycle (0800–2000 h) at 21 °C or 30 °C (thermoneutrality). The mice were fed either a chow diet (R70 Standard Diet, Lactamin AB, Vadstena, Sweden; 4.5 gm% fat, 14.5 gm% protein, 60 gm% carbohydrates) or a high-fat diet (HFD; D12451, Research Diets Inc., New Brunswick, NJ, USA; 24 gm% fat, 24 gm% protein and 41 gm% carbohydrate). At age 12 weeks, mice were randomly assigned to ether control or C_12_TPP-treated group. Both treated and control mice received food *ad libitum*. Each mouse in the pair-fed group was paired to a mouse from the treated group according to their body weight and received the same amount of food eaten by the corresponding treated mouse during the previous day. Pair-fed mice received one meal every 24 h at 2000 h to prevent disturbance of circadian rhythm.^14^ Water and food intake, as well as body weight, were measured always at the same time between 1900 and 2000 h. The C_12_TPP-treated mice were provided with water supplemented with one of two doses of C_12_TPP (5 or 50 μmol per (day•kg body weight)). Control and pair-fed mice received tap water supplemented with the corresponding dose of vehicle (NaBr and 0.07 to 0.1% ethanol, because C_12_TPP is a bromide salt dissolved in ethanol). The water was changed every 4 days. The body composition (body fat and lean mass) was measured with *in vivo* magnetic resonance imaging using an EchoMRI-100 instrument (EchoMRI LLC, Houston, TX, USA).

The animal protocols were conducted in accordance with the guidelines for the humane treatment of animals and were approved by the Animal Ethics Committee of the North Stockholm Region.

### Indirect calorimetry and energy expenditure

Indirect calorimetry was performed principally as described in Abreu-Vieira *et al.*^15^ using a Somedic INCA apparatus (Horby, Sweden). The resting metabolic rate (RMR) was calculated by determining the minimal stable (during at least 10–15 min) oxygen consumption rate during the light phase. The respiratory exchange ratio (RER) was calculated by determining the ratio of the rate of CO2 production to the rate of O2 consumption. The total energy expenditure (TEE) was calculated using two methods: (1) based on indirect calorimetry (TEEic) using the Weir equation: TEEic, kJ=(16.3 × volume O_2_+4.57 × volume CO_2_)/1000^(ref. 16)^ and (2) based on food intake and changes in body composition using the energy balance technique (TEEbal), kJ=energy intake (kJ)−39.33 × fat mass change (g)−4.184 × lean mass change (g)).^17^

### Fecal energy and lipid analysis

All feces produced by each mouse were collected, weighed and stored at −20 °C until being subjected to bomb calorimetry (Oxygen Bomb Calorimeter 6300 (Parr Instrument, Molin, IL, USA)) or a lipid analysis (gravimetric following a Folch extraction, as in Zadravec *et al.*^18^).

### Isolation and oxygen consumption of brown-fat mitochondria

Brown-fat mitochondria were isolated by differential centrifugation as described in Cannon *et al.*^19^ The oxygen consumption was measured with a Clark-type oxygen electrode (Yellow Springs Instrument Co., Yellow Springs, OH, USA) in a sealed chamber at 37 °C, as described previously.^20^ The mitochondria (0.25 mg protein per ml) were incubated in medium consisting of 125 mM sucrose, 20 mM K+-Tes (pH 7.2), 2 mM MgCl2, 1 mM EDTA, 4 mM KPi and 0.1% fatty-acid-free bovine serum albumin and 3 mM malate.

### Primary brown adipocyte culture and oxygen consumption

Primary brown adipocytes were cultured in medium supplemented with 1 μM rosiglitazone maleate (Alexis Biochemicals, San Diego, CA, USA) as described previously.^21^ C_12_TPP was added to brown adipocytes cultured for 7 days at a concentration of 1 μM and control cells were treated with 0.1% ethanol only. After a 3 h incubation, the cells were collected and the oxygen consumption was monitored with a Clark-type oxygen electrode as described previously.^22^

### Western blottings

Western blottings were principally performed as described in Shabalina *et al.*^23^ The levels of mitochondrial respiratory chain proteins (Complex I (NDUFB8), Complex II (SDHB), Complex III (UQCRC2), Complex IV (COX2) and Complex V (α-subunit *F*_o_*F*_1_-ATP-syntase) were examined using the Total OXPHOS Mouse Antibody cocktail from MitoSciences (#MS601, Eugene, OR, USA)) (diluted 1:15 000). UCP1 and voltage-dependent anion channel (VDAC) expressions were examined using either a UCP1 antibody produced in rabbit against the C-terminal UCP1 decapeptide at a dilution of 1:20 000 (in-house product) or a VDAC antibody from Cell Signaling (#4661S) diluted 1:2000. The immunoblottings were visualized in a charge-coupled device camera and expression was quantified using the Image Gauge V3.45 program (Fuji Film Co., Tokyo, Japan).

### Statistics

The detailed statistics is provided in Supplementary Information. KaleidaGraph 4.5.2 Synergy Software (Reading, PA, USA) and GraphPad Prism 6.0 (GraphPad Software, Inc., San Diego, CA, USA) were used for the graphs and statistical analysis. Mann–Whitney test was used for comparison of samples with *n*=4. Groups with normal distribution were compared with Student's two-tailed *t*-test (for comparing two groups), a one-way analysis of variance (for comparing more than two groups with equal variance) or a two-way analysis of variance (when comparing two parameters). Analysis of variance was followed by Bonferroni *post-hoc* test for multiple comparisons. All data were expressed as mean±s.e.m. Significance was accepted at the level of *P*<0.05 (indicated in the graph by one symbol), *P*<0.01 (two symbols) and *P*<0.001 (three symbols).

## Results

### C_12_TPP combats HFD-induced obesity in mice

**Figure 1:**
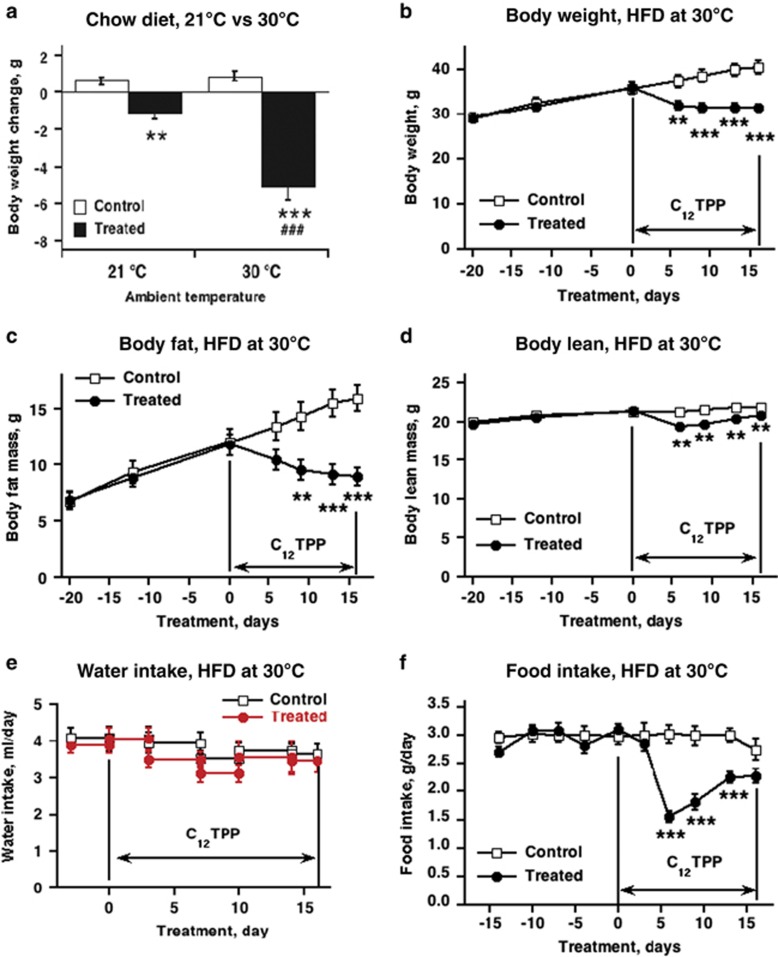
Body weight and food intake of wild-type mice treated with C_12_TPP. (**a**) Body weight change in mice maintained on a chow diet acclimated at 21 or 30 °C and treated with vehicle (control) or C_12_TPP (treated). The C_12_TPP dose was 50 μmol per (day•kg body weight) for 7 days. The values are the means±s.e.m. of five to six mice in each group. Student *t*-test for unpaired data with unequal variance was used for comparison. *Significant differences between the control and C_12_TPP-treated groups; ^#^significant difference between 21 and 30 °C. Time course of body weight (**b**), body fat mass (**c**), body lean mass (**d**), water (**e**) and food intake (**f**) of mice on a HFD before C_12_TPP treatment and during 16 days of C_12_TPP treatment. The period of treatment is indicated by arrows. The values are the means±s.e.m. (*n*=8 in each group). The statistical analysis of effects was conducted with a repeated measures 2-way analysis of variance (ANOVA): in **b**, **c** and **f** (time: *P*<0.001; treatment: *P*<0.001; interaction *P*<0.001); in **d** (time: *P*<0.01; treatment: *P*<0.001; interaction *P*<0.05). Asterisks in graphs indicate significant differences between the control and C_12_TPP-treated groups.

In the first experimental series, we tested the metabolic effects of C_12_TPP in C57Bl/6 mice consuming a standard chow diet and living at an ambient temperature of 21 °C. The drinking water was supplemented with 0.04 mM or 0.4 mM C_12_TPP; the water intake was ~4 ml per day, which resulted in C_12_TPP doses of 5 or 50 μmol per (day•kg body weight). One week of treatment with the high dose induced a minor but significant reduction in body weight (Figure 1a), whereas the low dose was ineffective (not shown).

The stimulation of metabolism by C_12_TPP at 21 °C may have been masked by increases in facultative thermogenesis due to the cold environment.^24, 25^ To explore this possibility, we tested the effects of C_12_TPP at 30 °C (thermoneutrality). At thermoneutrality, the body weight loss of mice treated with C_12_TPP was fourfold increased compared with that observed at 21 °C (Figure 1a). In all further experiments, the mice were maintained at 30 °C and the chow diet was exchanged for a HFD to induce obesity.

The time-dependent effect of C_12_TPP was studied in obese mice maintained on a HFD for 6 weeks before the C_12_TPP treatment and during the 16 days of treatment (Figures 1b–f). The body weights of control mice, which received vehicle, increased during the experiment, as expected. In contrast, C_12_TPP-treated mice displayed a remarkable decrease in body weight, which was observed as early as during the first 6 days of the treatment (Figure 1b). On the 16th day of the treatment, the treated mice lost 12% of the initial body weight. The decrease in body weight was paralleled by a decrease in body fat. The mice lost 24% of their body fat (Figure 1c), whereas body lean mass was only slightly affected (Figure 1d). Thus, C_12_TPP counteracts diet-induced obesity. Therefore, we continued to investigate the mechanism underlying this reduction in body weight/body fat by studying various parameters, such as food intake and energy expenditure.

### C_12_TPP reduces food intake

C_12_TPP did not affect water intake and water palatability (Figure 1e and Supplementary Figures S1a and b). However, C_12_TPP treatment significantly reduced food intake. Food intake was lowest (50% reduction) between the second and the sixth day of treatment and was spontaneously restored thereafter (Figure 1f). During the posttreatment period, food intake was normal and the body weight slowly reached the control level (Supplementary Figures S1c and d). Thus, the reduction in food intake contributes to the body weight loss observed in C_12_TPP-treated mice.

### C_12_TPP recruits BAT and uncouples brown-fat mitochondria and adipocytes via an UCP1-independent mechanism

**Figure 2:**
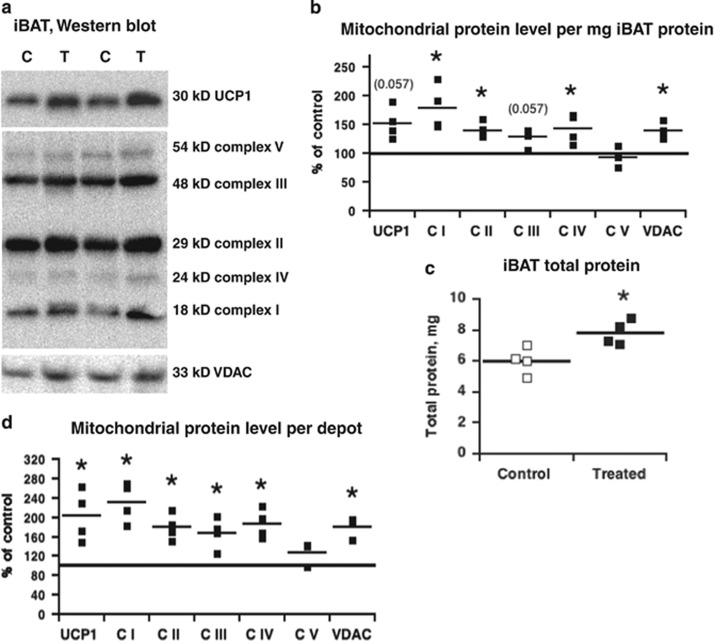
Recruitment of BAT in wild-type mice treated with C_12_TPP for 16 days on a HFD at thermoneutrality. (**a**) Western blotting of mitochondrial proteins performed on interscapular BAT (iBAT) protein extract. (**b**) Mitochondrial protein content per mg tissue protein taken from quantification of western blotting as in **a**. (**c**) Total iBAT protein content. (**d**) Mitochondrial protein content per iBAT depot. In **b**–**d**, the mean and individual data points of four independent tissue extracts of each group are presented. For graphic presentation on **b** and **d**, the mean protein level of control iBAT was defined as 100% and the levels in iBAT from C_12_TPP-treated mice expressed relatively to this value. For statistics on **b**–**d**, the raw data were analyzed with Wilcoxon–Mann–Whitney test. Asterisks indicate significant differences between the control and C_12_TPP-treated groups.

Brown adipose tissue (BAT) affects metabolic efficiency via non-shivering thermogenesis by combusting a fraction of the food, which attenuates body weight gain via the activity of UCP1.^26^ The total mitochondrial supplement and UCP1 content increased in the interscapular BAT of C_12_TPP-treated mice (Figure 2). Thus, mitochondria and UCP1 activity may be involved in the mechanism by which C_12_TPP reduces body weight in mice.

**Figure 3:**
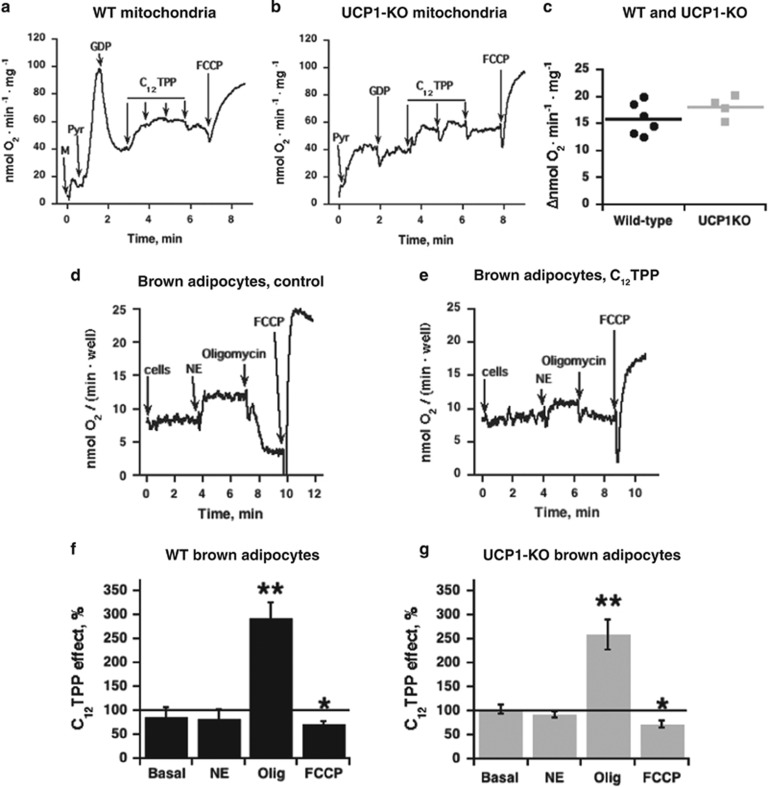
C_12_TPP-stimulated oxygen consumption in brown-fat mitochondria and brown adipocytes isolated from wild-type and UCP1-KO mice. Representative trace depicting titration with C_12_TPP of brown-fat mitochondria from wild-type (**a**) or UCP1-KO (**b**) mice. C_12_TPP was successively added at concentrations ranging from 11 to 44 μM (the concentration was increased by 11 μM in each step). Additions were 5 mM pyruvate (*Pyr*), 1 mM GDP and 2.1 μM carbonyl cyanide *p*-(trifluoromethoxy) phenylhydrazone (FCCP). (**c)** Comparison of the effects of C_12_TPP in wild-type and UCP1-KO brown-fat mitochondria. The mitochondria were examined as shown in **a** and **b**, and the values were obtained by estimating the maximal responses. The mean and individual data points of four to six independent mitochondrial preparations for each group are presented. Representative traces depicting the oxygen consumption of control (**d**) and C_12_TPP-pretreated (**e**) brown adipocytes. Primary cultures of brown adipocytes were treated with 0.1% ethanol (control) or 1 μM C_12_TPP for 3 h before harvesting. Additions were 1 μM β-adrenergic agonist norepinephrine (NE), 2 μM oligomycin and 60 μM FCCP. C_12_TPP effects on oxygen consumption rates in brown adipocytes originating from wild-type mice (**f**) or UCP1-ablated mice (**g**). Control and C_12_TPP-treated adipocytes were examined in parallel as shown in **d** and **e**. The oxygen consumption rate of control adipocytes on a given day was defined as 100% and oxygen consumption rate of C_12_TPP-treated adipocytes from parallel examination is expressed relative to that of these control adipocytes (as % of basal, NE, oligomycin and FCCP). The bars represent the means±s.e.m. of five independent cell cultures of each genotype. The raw data were analyzed with Student's *t*-test and asterisks indicate significant differences between control and C_12_TPP-treated cells.

In a series of *in vitro* experiments, we examined the ability of C_12_TPP to activate isolated brown-fat mitochondria and brown adipocytes (Figure 3). *In situ*, the innate activity of UCP1 is inhibited by high cellular levels of purine nucleotides.^27, 28^ To mimic this *in situ* condition, we first inhibited respiration with high amounts of the UCP1 inhibitor GDP and then added C_12_TPP. C_12_TPP re-activated UCP1-containing mitochondria (Figure 3a). To examine whether this C_12_TPP effect was mediated by UCP1, we examined the effects of C_12_TPP in UCP1 knockout (KO) mitochondria (Figure 3b). In contrast to the wild-type mitochondria (Figure 3a), pyruvate-supported respiration was low in brown-fat mitochondria lacking UCP1 and the addition of GDP did not affect respiration (Figure 3b). These results are consistent with Matthias *et al.*;^29^ Monemdjou *et al.*^30^ However, the C_12_TPP concentration–response curves were similar in wild-type and UCP1-KO mitochondria; maximal responses were observed at the same C_12_TPP concentration (22 μM). The magnitudes of these responses were the same in wild-type and UCP1-KO mitochondria (Figure 3c). Thus, remarkably, C_12_TPP can activate brown fat mitochondria. However, the mechanism of activation was different from that associated with the classical activators of UCP1 fatty acids. The concentration–response curves of the structural analogue of the C_12_TPP tail, lauric acid, clearly differed between wild-type and UCP1-KO mitochondria (Supplementary Figure 2); these results are consistent with Shabalina *et al.*^28, 31^

To test the ability of C_12_TPP to activate cellular thermogenesis, we examined the effects of C_12_TPP in brown adipocytes in culture (Figures 3d–g). The basal and norepinephrine-stimulated oxygen consumption rates were the same in the control and C_12_TPP-treated wild-type brown adipocytes (Figures 3d and e). However, when mitochondrial oxidative phosphorylation was inhibited by oligomycin, the oxygen consumption of C_12_TPP-treated brown adipocytes was considerably increased compared with control adipocytes (Figure 3f), which reflects increased mitochondrial membrane proton conductance in the treated cells. The same pattern was also observed in UCP1-KO brown adipocytes (Figure 3g).

Thus, we concluded that C_12_TPP induced uncoupling in isolated brown-fat mitochondria and intact brown adipocytes in an UCP1-independent manner.

The level of this uncoupling was mild and did not significantly inhibit or disrupt mitochondrial oxidative capacity. The artificial protonophore carbonyl cyanide *p*-(trifluoromethoxy) phenylhydrazone applied to mitochondria after C_12_TPP exerted a high stimulatory effect (Figures 3a and b), indicating that the mitochondria retained their high oxidative capacity. In cells, the carbonyl cyanide *p*-(trifluoromethoxy) phenylhydrazone response was only slightly affected (Figures 3f and g).

**Figure 4:**
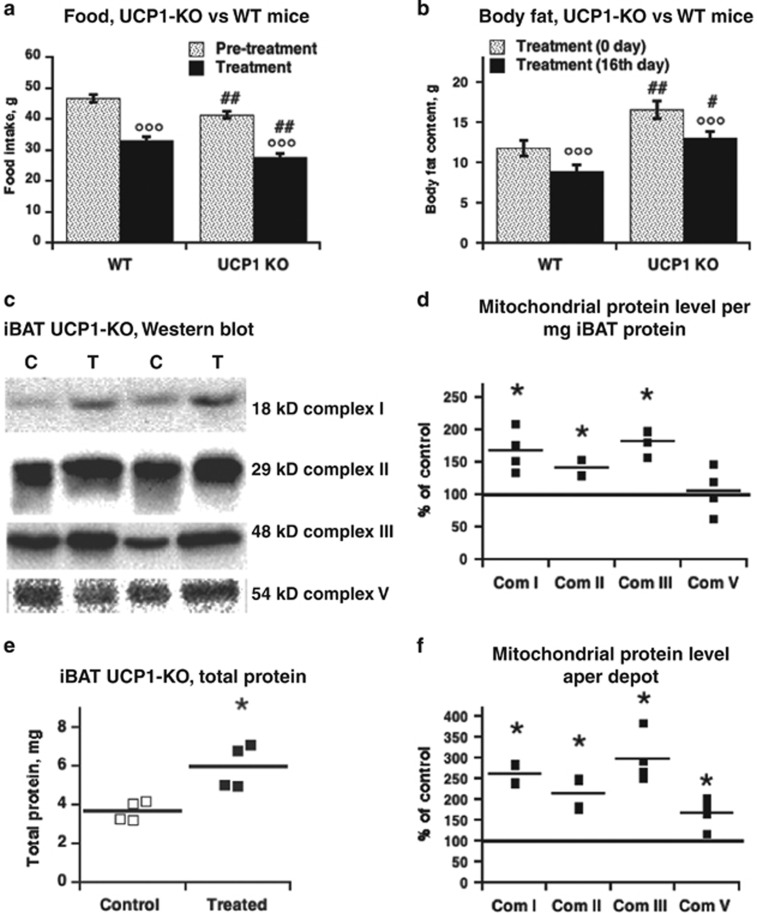
UCP1-independent effects of C_12_TPP in mice. (**a**) Food intake for 16 days of pretreatment and treatment period in the wild-type and UCP1-KO mice on HFD at thermoneutrality. The data for wild-type mice were obtained from Figure 1f. (**b**) Body fat mass on day 0 (the start of C_12_TPP treatment) and on the 16th (last) day of treatment in wild-type and UCP1-KO mice. The data for wild-type mice were obtained from Figure 1c. In **a** and **b**, the values are means±s.e.m. (*n*=8 in each group). The data were statistically analyzed with a two-way mixed analysis of variance (ANOVA) with treatment as within-subjects factor and genotype as between-subjects factor: in **a**, (genotype: *P*<0.01; treatment: *P*<0.001; interaction *ns*); in **b**, (genotype: *P*<0.01; day: *P*<0.001; interaction *ns*). °Significant differences between days (pretreatment and treatment, or day 0 and day 16); #Significant differences between genotypes. (**c**) Western blotting of mitochondrial proteins in the total interscapular BAT (iBAT) protein extract from UCP1-KO mice treated with C_12_TPP for 16 days on a HFD at thermoneutrality. (**d**) Mitochondrial protein content per mg tissue extract taken from quantification of western blotting as in **c**. (**e**) iBAT total protein content. (**f**) Mitochondrial protein content per total iBAT depot. In **d**–**f**, the mean and individual data points of four independent tissue extracts of each group are presented. For graphic presentation on **d**–**f**, the mean protein level of control iBAT was defined as 100% and the levels in iBAT from C_12_TPP-treated mice expressed relatively to this value. For statistics on **d**–**f**, the raw data were analyzed with Wilcoxon–Mann–Whitney test. Asterisks indicate significant differences between the control and C_12_TPP-treated groups.

Although we demonstrated that the *in vitro* uncoupling effect of C_12_TPP in isolated mitochondria and cultured brown adipocytes was independent of the presence of UCP1, we could not exclude the possibility that the effects of C_12_TPP *in vivo* were mediated by UCP1. For example, a reduction in food intake may depend on UCP1, as recently demonstrated.^32^ Therefore, we compared food intake in wild-type and UCP1-KO mice. The C_12_TPP-induced reduction in food intake in UCP1-KO mice was the same as that observed in wild-type mice (Figure 4a and Supplementary Figure S3a), which strongly suggests that C_12_TPP affected food intake independently of the presence of UCP1. However, UCP1-KO mice generally ate less than wild-type mice (Figure 4a). Despite this lower food intake, UCP1-KO mice were significantly fatter than wild-type mice (Figure 4b), as demonstrated in Feldmann *et al.*^24^ Importantly, the C_12_TPP-induced decreases in body weight and fat weight were similar in UCP1-KO and wild-type mice (Figure 4b and Supplementary Figures S3b and c). Changes in lean body mass were also independent of UCP1 (data not shown).

The increases in the levels of total interscapular BAT protein and individual mitochondrial protein complexes (Figures 4c–f) were similar in UCP1-KO and wild-type mice (Figure 2).

BAT is a powerful tissue for releasing energy as heat.^33^ Furthermore, BAT can contain a high level of endogenous fatty acids under physiological conditions, which favors C_12_TPP uncoupling by facilitating the cycling of endogenous fatty acids in mitochondria.^10^ Skeletal muscle is another metabolically active tissue that contributes to energy expenditure. However, C_12_TPP did not affect the mitochondrial protein content in the skeletal muscle of mice treated with C_12_TPP (Supplementary Figure S4), which supports the hypothesis that BAT may have an important role in the mechanism of C_12_TPP.

### The anti-obesity effect of C_12_TPP is partially attributable to decreased food intake

**Figure 5:**
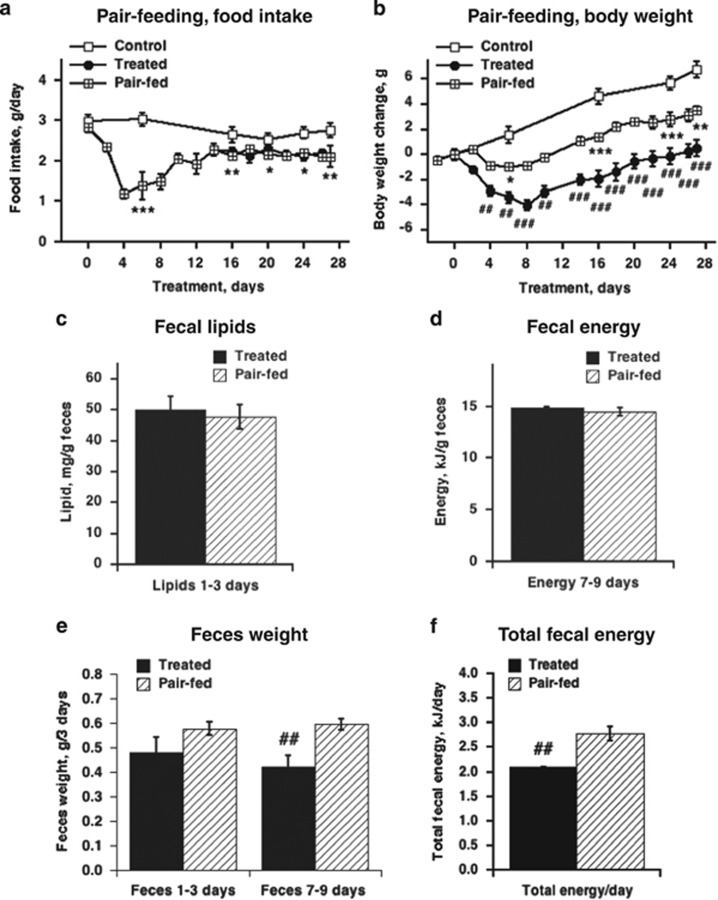
Food intake-independent effects of C_12_TPP in wild-type mice on a HFD at thermoneutrality. Time course of food intake (**a**) and body weight change relative to day 0 (**b**) of control, C_12_TPP-treated mice and mice pair-fed with the treated group. Treatment and pair-feeding started on day 0 and finished on day 27. The values are the means±s.e.m. of six to seven mice per group. The effects for overlapping time points with the control were statistically analyzed with a repeated measures two-way analysis of variance (ANOVA): for both **a** and **b** (time: *P*<0.0001; treatment: *P*<0.0001; interaction *P*<0.001). The effects for overlapping time points for treated and pair-fed mice was statistically analyzed with a repeated measures two-way ANOVA for **a**: the food intake in pair-fed group was pre-defined and cannot be included in the ANOVA; for **b** (time: *P*<0.0001; treatment: *P*<0.0001; interaction *P*<0.001). *Significant differences between the pair-fed and control groups. #Significant differences between the pair-fed and treated groups. The difference between the control and treated groups is not shown. (**c** and **d**) Lipid and energy contents of feces. A lipid analysis (**c**) was performed on feces from the first 3 days of treatment and bomb calorimetry was used to estimate the energy (**d**) content of feces from 7 to 9 days of treatment. Bars represent the mean±s.e.m. of four to six mice per group. (**e**) Amount of feces produced during the first three days (1 to 3) and 7 to 9 days of treatment. Bars represent the mean±s.e.m. of five to six mice per group. The data were statistically analyzed with a repeated measures two-way ANOVA: (treatment: *P*<0.001; days *ns*; interaction *ns*). #Significant differences between the pair-fed and treated groups. (**f**) Total fecal energy per day of C_12_TPP treatment. The fecal energy (as in **d**) was multiplied by the amount of feces (as in **e**) and this amount was divided by 3 days. Bars represent the mean±s.e.m. of five to six mice per group. The data were analyzed with Student's *t*-test and # indicates significant differences between the pair-fed and treated groups.

To estimate the extent to which the effects of C_12_TPP were determined by a decrease in food intake, we introduced a pair-fed group of mice and increased the duration of the treatment for up to 27 days. Obvious differences were observed in the body weights of C_12_TPP-treated and pair-fed mice (Figures 5a and b). When food intake was lowest (between the fourth and the eighth day of treatment), C_12_TPP-treated mice lost threefold more body weight than pair-fed mice (Figure 5b). Thus, the C_12_TPP-induced loss in body weight is due to two mechanisms: food intake dependent and food intake independent. The food intake-dependent component contributes to a maximum of 30% of the body weight loss. Thus, most of the body weight loss must be explained by another mechanism. To highlight the impact of food intake-independent mechanisms, we selected the pair-fed group as the proper control for the C_12_TPP-treated group. All further parameters were compared between treated and pair-fed mice.

The effect of C_12_TPP on body weight may be attributed to an adverse effect on the intestines, for example, impaired digestion and/or food absorption. However, a pathomorphological examination of the alimentary system did not reveal any sign of digestive tract impairment in C_12_TPP-treated mice (data not shown). The energy content and amount of lipid per gram of feces were also similar in treated and pair-fed mice (Figures 5c and d). Importantly, both the amount of feces and the total fecal energy were reduced in treated mice (Figures 5e and f). From the seventh to the ninth day of treatment, treated mice excreted 5.9±0.4% of the energy consumed, whereas pair-fed mice excreted 8.2±0.4% (*P*<0.01).

Thus, C_12_TPP simultaneously reduces food intake and excreted energy, which together explain ~20–30% of mouse body weight loss.

### High RMR in C_12_TPP-treated mice

**Figure 6:**
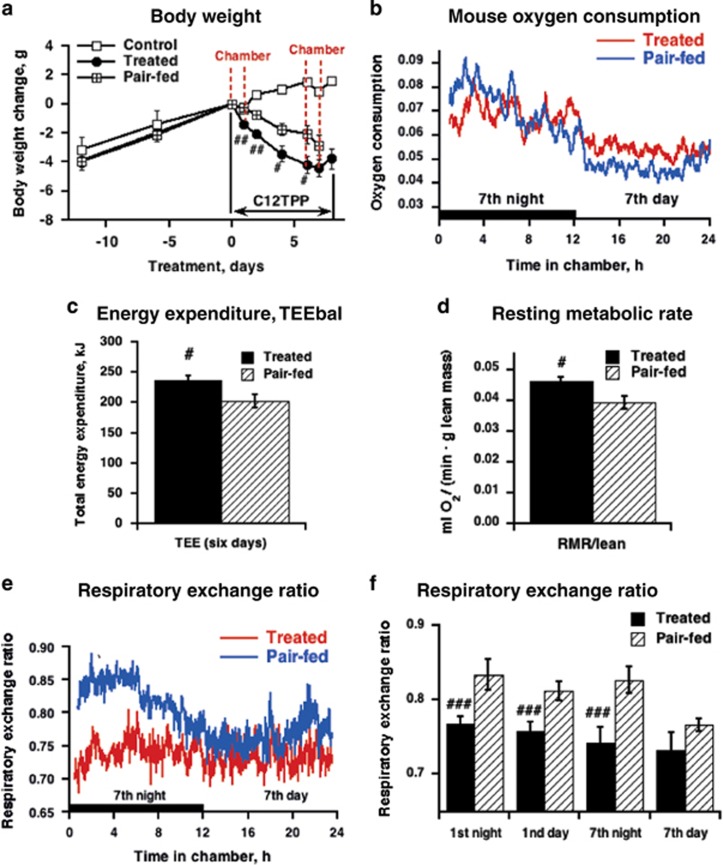
Effects of C_12_TPP on the rates of oxygen consumption and RER in wild-type mice on a HFD at thermoneutrality. (**a**) Change in body weight over time relative to day 0 in control, C_12_TPP-treated and pair-fed to treated mice. Treatment started on day 0 and finished on day 7, as is indicated by arrows. The mice were exposed twice to indirect calorimetry measurements on day 1 and 7 of treatment. The periods inside the chamber are indicated by red lines. The values are the means±s.e.m. of five to six mice per group. The effects were statistically analyzed using a repeated measures two-way analysis of variance (ANOVA) (time: *P*<0.01; treatment: *P*<0.01; interaction *P*<0.01). #Significant differences between the treated and pair-fed groups. Significant differences from the control are not shown. (**b**) Rate of oxygen consumption (ml O_2_ per min**·**g lean body mass) of treated and pair-fed mice on the seventh day of treatment. Nighttime is indicated by the black box on the *x* axis. The values are the means of six mice per group. (**c**) TEE (TEEbal) estimated using the energy balance method for the 6 days of treatment. (**d**) RMR in C_12_TPP-treated and pair-fed mice during the seventh light phase of day of treatment. In **c** and **d**, the values are the means±s.e.m. (*n*=6 for each group). The data were analyzed with a paired Student's *t*-test and the # indicates significant differences between the pair-fed and treated groups. (**e**) RER traces during the seventh day of treatment. Each trace is the mean of *n*=6 mice per group. (**f**) Average RER of treated and pair-fed mice. The values are the means±s.e.m. (*n*=5–6 for each group). The effects were statistically analyzed with a two-way ANOVA matched for both time and mouse (separate for day 1 and day 7 due to not being the same animals): day 1 (time: *P*<0.05; treatment: *P*<0.05; interaction *P*<0.05); day 7 (time: *P*<0.05; treatment: *P*<0.05; interaction *P*<0.01). #Significant differences between the treated and pair-fed groups.

Indirect calorimetry was applied to examine the oxygen consumption, TEE (TEEic), RMR and RER. The mice were placed into indirect calorimetry chambers during the first and the seventh day of treatment (Figure 6a). The oxygen consumption rates were higher during dark phase than during the light phase (Figure 6b), reflecting circadian rhythm of mice.

The TEEic did not differ between the treated and pair-fed group (Supplementary Table). However, the TEE estimated over 6 days using an energy balance method^17^ (TEEbal) was 15% increased in treated compared with pair-fed mice (Figure 6c). Surprisingly, these two methods of TEE estimation yielded different results. The TEEbal was measured at standard conditions in the ‘home cage,' which provided an accurate integrated long-term measurement of energy expenditure, whereas the TEEic may have been affected by potentially confounding stress that may accompany the use of an indirect calorimetry system.^17^ Pair-fed mice lost more body weight in the indirect calorimetry chamber compared with a standard environment, whereas C_12_TPP-treated mice were minimally affected by the new environment (Supplementary Figure S5a).

Basal mitochondrial proton leakage (uncoupling) significantly contributes to the RMR in mice.^7, 34^ To identify the uncoupling activity of C_12_TPP *in vivo*, we determined the RMR of mice treated with C_12_TPP. The RMR per mouse was 6% (day 1) and 11% (day 7) increased in C_12_TPP-treated mice compared with pair-fed mice (Supplementary Table). The normalization of the RMR by the lean body mass^35^ (Supplementary Figure S5b) resulted in an 18% increased RMR in treated mice compared with pair-fed mice (Figure 6d).

Thus, both an increased RMR and reduced energy intake could explain the anti-obesity activity of C_12_TPP. Importantly, a comparison with pair-fed animals minimizes the impact of the thermic effect of food on the RMR and consequently enables the estimation of the uncoupling activity of C_12_TPP *in vivo.*

### C_12_TPP treatment enhances fatty acid utilization

The RER of pair-fed mice was ≈0.85 and slightly higher during the dark than during the light phase (Figures 6e and f). The RER of the C_12_TPP-treated mice was significantly reduced compared with pair-fed mice, which was noted as early as the first day of the treatment and remained low until the seventh night (Figure 6f). In the latter case, C_12_TPP decreased the RER to a minimal level (0.7), indicating a total switch to lipid catabolism on C_12_TPP treatment. Thus, C_12_TPP treatment enhanced fatty acid utilization via a mechanism independent of decreases in food consumption.

## Discussion

### C_12_TPP induces mitochondrial uncoupling

In this study, we demonstrated the uncoupling effects of C_12_TPP in isolated brown-fat mitochondria, brown adipocyte culture and in mice *in vivo*. Although these effects were independent of UCP1, the effect of C_12_TPP on the metabolic rate may be mediated by brown-fat mitochondria. Free fatty acids are released in BAT on adrenergic stimulation. C_12_TPP may facilitate the flip-flopping of fatty acid anions in the mitochondrial membrane, which dissipates the energy of the transmembrane potential. This mechanism may be additionally stimulated by the observed upregulation of mitochondrial protein synthesis in the BAT on C_12_TPP treatment. Interestingly, this effect of C_12_TPP contrasts the effect of the classical uncoupler DNP, which reduces the thermogenic capacity of BAT.^25^ C_12_TPP does not affect skeletal muscle mitochondriogenesis, which is also in the contrast to the effect of DNP.^36^

The effect of C_12_TPP on body weight was more evident at thermoneutrality than 21 °C. The anti-obesity effects of the artificial uncoupler DNP and natural uncoupler UCP1 were strongly dependent on environmental temperature.^24, 25, 37, 38^ Thus, the present study confirms a general principle—the potential anti-obesity agents should be studied at thermoneutrality in mice. A recent study of the anti-obesity effect of a mitochondria-targeting mitoQ, which can uncouple mitochondrial respiration *in vitro*,^39^ did not reveal an increase in the metabolic rate of mitoQ-treated mice.^40^ However, the study was performed in mice maintained at 22 °C, which could explain the lack of an effect of mitoQ on the metabolic rate of the mice.

The C_12_TPP-induced increase in metabolic rate was small. It is equal to the change in metabolism rate, which occurs in mice on change of only 3 °C of ambient temperature.^33^ Such a small increase in metabolism should not detrimentally affect the function of the cardiovascular, renal and endocrine systems. It has been demonstrated that the slightly higher metabolism observed after exercise or after short exposure to cold ameliorates the symptoms of diabetes and atherosclerosis, and reduces the risk of cardiovascular diseases.^41, 42, 43, 44, 45^ Thus, a beneficial effect of C_12_TPP on these pathologies may also be proposed.

### C_12_TPP enhances fatty acid utilization

C_12_TPP significantly reduced the RER, which indicates enhanced fatty acid utilization in mice. Several mechanisms are hypothesized to underlie this enhanced fatty acid utilization. The first hypothesis relies on the suggestion that mitochondrial uncoupling generally increases mitochondrial fatty acid oxidation, which ultimately results in a low RER throughout the body. Natural uncoupling by UCP1 in brown/brite adipose tissues typically correlates well with enhanced mitochondrial fatty acid utilization and lipid droplet remodeling.^23, 41, 46^ Furthermore, the mild mitochondrial uncoupler niclosamide ethanolamine reduced the RER consistently with a significant reduction in adipose tissue depots.^47^

Enhanced fatty acid utilization is also related to increased lipid availability in the systemic circulation,^48, 49^ improved fatty acid uptake into cells and/or the enhanced transport of fatty acid-derived substrates into mitochondria.^50, 51^ The C_12_TPP cation might facilitate the delivery of fatty acid anions to fatty acyl-CoA synthetases, which is an attractive prospect.^52^

The effect of C_12_TPP on RER was large and under resting condition, it could be entirely explained by the utilization of fatty acids in BAT. Several facts favorite this hypothesis: (1) C_12_TPP uncouples mitochondria by means of endogenous fatty acids ^10^ and BAT is the only tissue enriched by both free fatty acids and mitochondria;^33^ (2) C_12_TPP recruits BAT; and (3) BAT actively controls the plasma triglycerides clearance.^41^ However, under strenuous exercise, a fatty acid utilization is highly increased in skeletal muscle^53^ and whether C_12_TPP could facilitate this process is currently unknown.

### C_12_TPP affects mouse appetite

C_12_TPP potentially reduced food intake in mice by affecting the production (or functioning) of hormone-like compounds that affect appetite. Indeed, the effects of C_12_TPP on food intake resemble the effects of the anorexic gut peptide YY.^54^ Development of tolerance to appetite suppression appears to be a common feature of many anti-obesity drugs: for example, rimonabant, sibutramine and tesofensine (reviewed in Fernstrom *et al.*^55^).

Other mitochondria-targeting compounds such as mitoQ^40^ and C_4_R1^(ref. 12)^ also influence food intake. The ability of all three mitochondria-targeting compounds that have been studied to influence appetite is intriguing; however, the mechanism underlying this influence remains elusive. Nevertheless, the direct action of mitoQ on the satiety center in the central nervous system has been excluded, because mitoQ does not enter the central nervous system.^56^

### C_12_TPP is a potential therapeutic route to combat obesity

Three anti-obesity effects of C_12_TPP were observed: decreased energy intake, elevated RMR and an increased utilization of fatty acids. This combination of several modes of action appears to be a desirable feature of C_12_TPP as an anti-obesity agent, because a poly-therapeutic strategy against obesity (targeting different metabolic pathways) often yields better results than strategies that modify one pathway.^1^

C_12_TPP did not affect water intake or the palatability and morphology of the alimentary tract. Treatment with C_12_TPP (27 days in total) also did not induce visible toxic effects in mice. Mice behavior was normal, even during periods of substantially reduced food intake. This C_12_TPP study was performed under humanized conditions (that is, human metabolism was modeled in mice), namely at thermoneutrality and with high fat and high sugar diets resembling obesity-inducing Western diets. Therefore, the anti-obesity properties of C_12_TPP are expected to be reproduced in man.

Thus, C_12_TPP appears to be promising as an effective and safe anti-obesity drug.
