# Supplementary material for: Mitochondria-targeted dodecyltriphenylphosphonium (C12TPP) combats high-fat-diet-induced obesity in mice
Source: Int J Obes (Lond). 2016 Sep 20;40(12):1864–74. doi: 10.1038/ijo.2016.146 (PMC5144127; doi:10.1038/ijo.2016.146)
Supplement: Supplementary Information [file ijo2016146x1.doc]

**Statistics (detailed)**

Western blot, bomb calorimetry, cellular oxygen consumption and pathomorphological examination of the alimentary system were performed blindly on coded samples. For measurement of body weight and other physiological parameters, as well as for tissues collection, control and treated animals were taken in random order.

KaleidaGraph 4.5.2 Synergy Software (Reading, PA, USA) and GraphPad Prism 6.0 (San Diego, CA, USA) were used for the graphs and statistical analysis. Mann-Whitney test was used for comparison of samples with n equal 4. Shapiro-Wilk test and skewness were used for assessment of normal distribution. Similarity of variances in different groups was compared using a F-test (HO: equal variance). Groups with normal distribution were compared with Student's two-tailed *t*-test (for comparing 2 groups), a 1-way analysis of variance (ANOVA) (for comparing more than 2 groups with equal variance) or a repeated measures 2-way ANOVA (for comparing more than 2 groups with equal variance and the effects of two parameters). ANOVA was followed by Bonferroni post hoc test for multiple comparisons. All data were expressed as mean ± s.e.m. Significance was accepted at the level of p < 0.05 (indicated in the graph by one symbol, p < 0.01 (two symbols) and p < 0.001 (three symbols).

Power analysis and sample size estimation were performed using Stata 14 (StataCorp LP, Texas, USA). The experiment in Figure 1a was a pilot experiment. For other experiments, the sample size (number of animals in the group) was determined based on the coefficient of variation of the values in Figure 1a (30°C) (10%), specified significance level (5%), and chosen power as 85% and desired effect of C_12_TPP as 15 %. The output of the power analysis was 8 animals in each group; this *n* value was used in the first experimental set-up. The observed effect of C_12_TPP in the first experiment was higher (25%) and for the estimation of the number of animals in the second experimental set-up, the power was chosen as 98-99%. The output of this power analysis was 5-6 animals in each group.

**Supplementary Table**.

**Total daily energy expenditure (TEEic) and resting metabolic rate (RMR) in C_12_TPP-treated and pair-fed mice**

The values are the mean ± SE of n=6 in each group of mice. 1-way ANOVA was used for multiple comparison statistics for all groups: (TEEic day 1, *P*=0.02); (TEEic day 7, *P*=0.04); (RMR day 1, *P*=0.02); (RMR day 7, *P*=0.05). For comparison between treated and pair-fed mice paired t-test was used. *: statistically significant differences between pair-fed and control group; #: statistically significant differences between pair-fed and treated groups; ° tendency to statistically significant differences between pair-fed and treated groups (*P*=0.06).

| Parameter | Day of treatment | Control | Treated | Pair-fed | % Treated *vs*  Pair-fed (100%) |
| --- | --- | --- | --- | --- | --- |
| TEEic  kJ/ 24 h/ mouse | 1^st^ | 35.2±0.8 | 31.8±0.9 | 32.9±0.6 | 97±2 |
|  | 7^th^ | 33.6±1.8 | 28.9±0.7 | 29.8±1.2 | 97±3 |
| RMR  ml O_2_/ min/ mouse | 1^st^ | 0.84±0.03 | 0.76±0.03^#^ | 0.71±0.02* | 106±2 |
|  | 7^th^ | 0.82±0.04 | 0.81±0.02**°** | 0.73±0.02 | 111±4 |

Pair-feeding decreased RMR as compared with vehicle control at day 1 and there was a tendency at day 7, reflecting impact of the thermic effect of feeding (the energy expended on the absorption and processing of nutrients).
