# Supplementary material for: Mitochondria-targeted dodecyltriphenylphosphonium (C12TPP) combats high-fat-diet-induced obesity in mice
Source: Int J Obes (Lond). 2016 Sep 20;40(12):1864–74. doi: 10.1038/ijo.2016.146 (PMC5144127; doi:10.1038/ijo.2016.146)
Supplement: Supplementary Figure S1 [file ijo2016146x2.pdf]

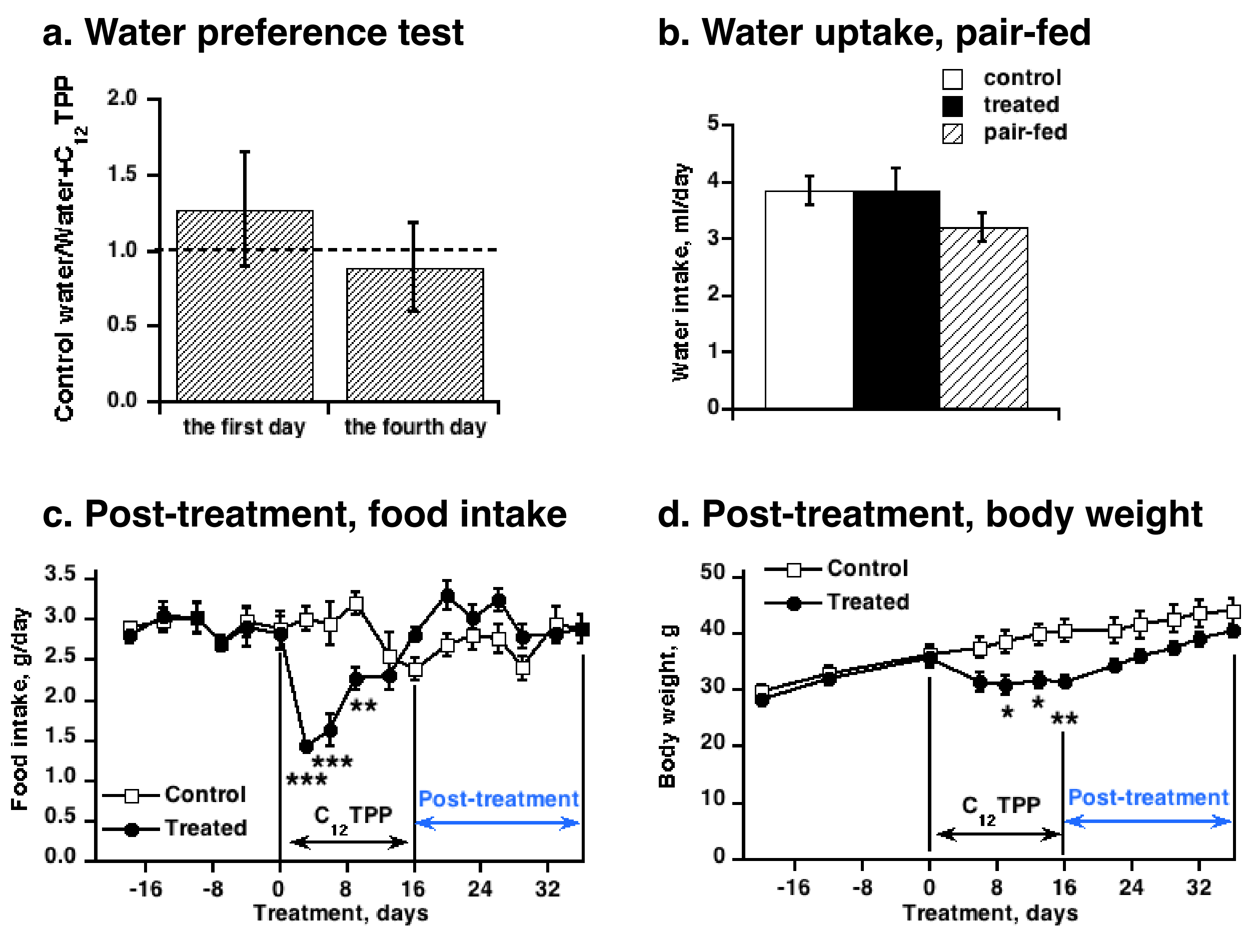

Supplementary Figure S1. Water preference test and water intake in wild-type mice treated with $C_{12}TPP$

(**a**) Water preference test in wild-type mice acclimated at 30°C. Since $C_{12}TPP$ was administrated in drinking water, a concern existed that it could influence the palatability of the water and reduce water intake, which could result in food aversion and body weight loss. We therefore performed a simple water preference test: wild-type mice received two bottles simultaneously – one bottle contained control water (with sodium bromide and ethanol), another bottle contained water supplemented with $C_{12}TPP$ (dose 50 μmol/(day • kg body weight)), and the mice thus could drink what they preferred. Volume of water consumed from each bottle was measured twice – after one day and after four days, and the ratio of control water intake to intake of water supplemented with $C_{12}TPP$ was calculated. The values are the means ± S.E. of 3 mice. The data were analyzed with Student's t-test.

(**b**) Water intake. Treatment with $C_{12}TPP$ at a dose of 50 μmol/(day • kg body weight) and pair-feeding were performed for 7 days in wild-type mice maintained at 30°C and on a HFD. The values are the means ± S.E. of 14 to 17 mice in each group. Data were combined from three independent experiments. The statistical analysis was conducted with an 1-way ANOVA.

(**c**, **d**) Time course of food intake (**c**) and body weight (**d**) in mice maintained on a HFD and at 30°C before $C_{12}TPP$ treatment, during 16 days of the treatment and during 20 days after the treatment. Four mice from group of eight mice presented on Figures 1 b-f were sacrificed on day 16 and tissues were analysed (Figure 2). In the group of remained four mice, food intake and body weight were measured during additional 20 days of the post-treatment period. The results are presented here. The values are the means ± S.E. of 4 mice in each group. The statistical analysis of effects was conducted with a 2-way ANOVA in **c**, (time: $P < 0.001$; treatment: $P < 0.05$; interaction $P < 0.001$); in **d**, (time: $P < 0.001$; treatment: $P < 0.001$; interaction ns). Asterisks in graphs indicate significant differences between the control and $C_{12}TPP$-treated groups.

a. Lauric acid titration

b. Concentration response curve

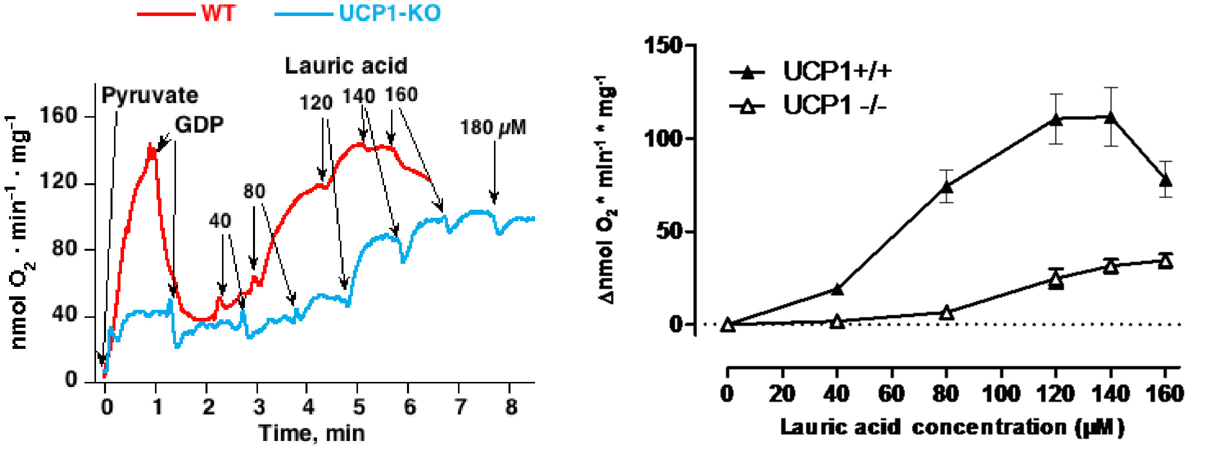

Supplementary Figure S2. The effect of lauric acid in brown-fat mitochondria isolated from wild-type and UCP1-KO mice

(a) Representative traces depicting titration with lauric acid of brown-fat mitochondria from wild-type and UCP1-KO mice. Lauric acid was successively added at concentrations ranging from 40 to 180 μM. The medium was also supplemented with 5 mM pyruvate and 1 mM GDP.

(b) Concentration-response curves of lauric acid in brown-fat mitochondria from wild-type and UCP1-KO mice. Mitochondria were examined as shown in a. The statistical analysis of effects was conducted with a 2-way ANOVA (genotype: $P < 0.01$; concentration: $P < 0.01$; interaction: $P < 0.01$). The points are the means ± S.E. of 6 independent mitochondrial preparations for each group.

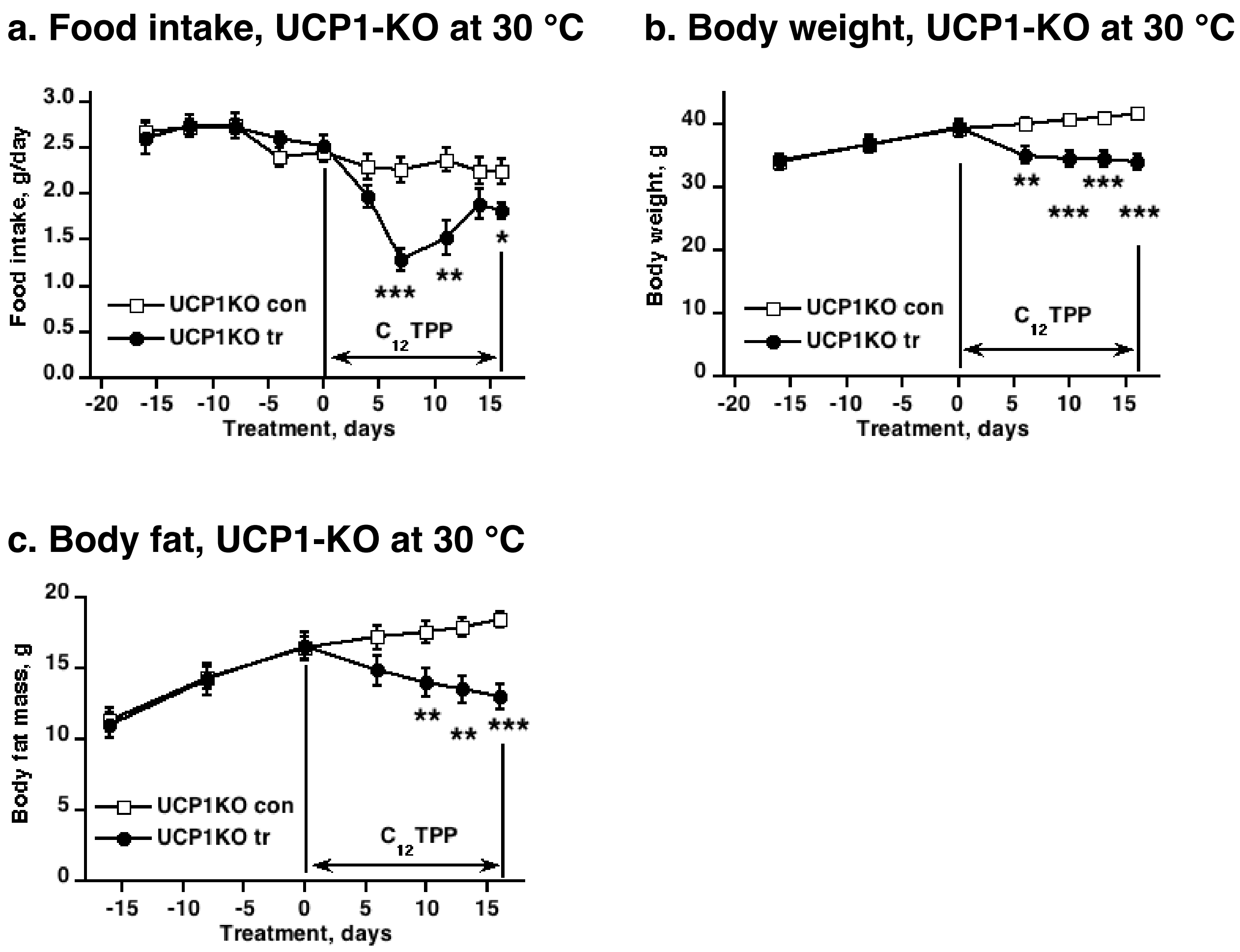

Supplementary Figure S3.

Time course of food intake (**a**), body weight (**b**) and body fat (**c**) of UCP1-KO mice maintained on a HFD and at 30°C and treated with C_{12}TPP. Treatment period is indicated by arrows. The values are the means ± S.E. of 8 mice in each group. The statistical analysis of effects was conducted with a 2-way ANOVA: in **a**, (time: $P < 0.001$; treatment: $P < 0.01$; interaction $P < 0.01$), in **b**, (time: $P < 0.001$; treatment: $P < 0.001$; interaction $P < 0.001$) and in **c**, (time: $P < 0.001$; treatment: $P < 0.01$; interaction $P < 0.01$). Asterisks in graphs indicate significant differences between the control and C_{12}TPP-treated groups.

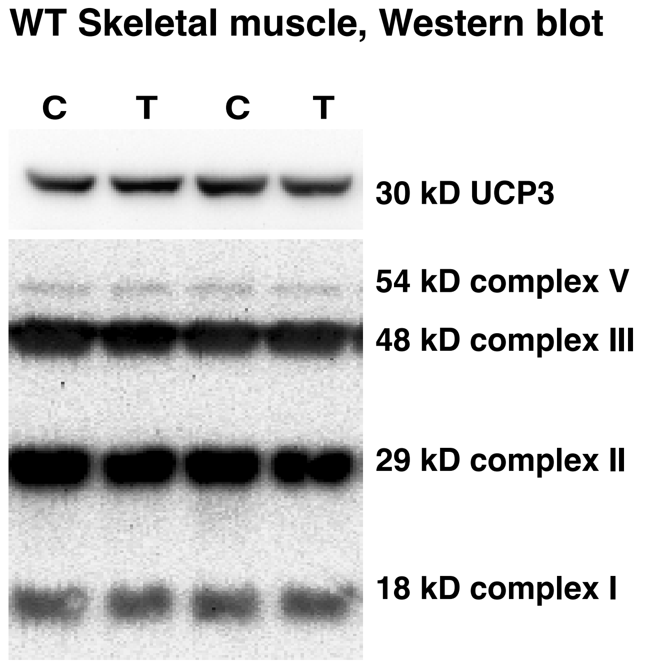

Supplementary Figure S4.

Western blot of mitochondrial proteins performed on skeletal muscle (gastrocnemius) tissue protein extract from wild-type mice maintained at 30°C and on a HFD and treated with vehicle (control indicated as **C**) or C_{12}TPP during 16 days (treated indicated as **T**).

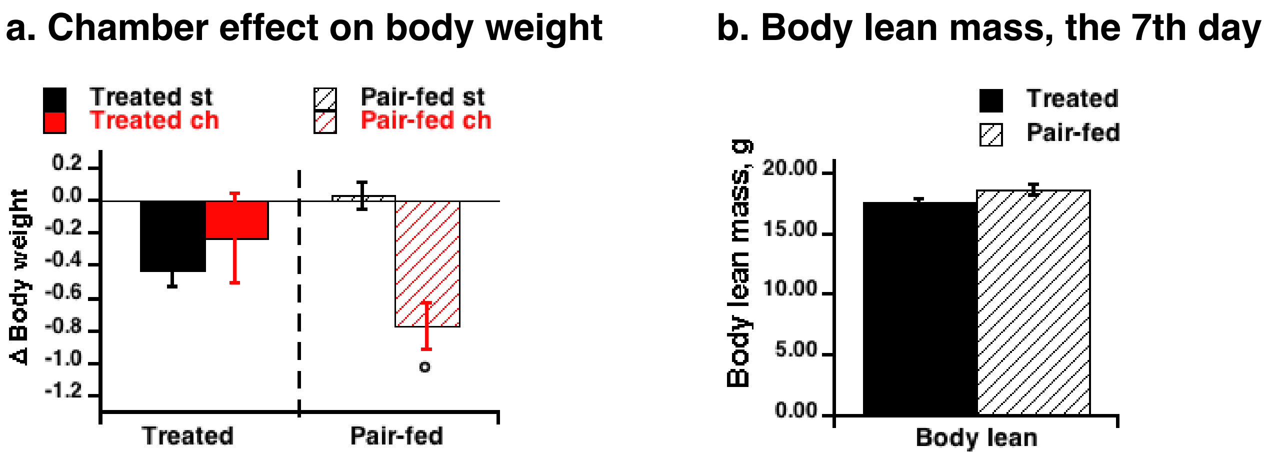

Supplementary Figure S5. Body weight changes and lean body mass on the day 7 of C_{12}TPP treatment

(**a**) Comparison of body weight change (on day 7 of C_{12}TPP treatment) in standard environment (animal house room) and inside indirect calorimetry chamber. The values are the means ± S.E. of 6 to 7 mice in each group. The data were statistically analysed with a 2-way ANOVA (treatment: ns; location: ns; interaction: $P < 0.01$). The symbol (°) indicates significant differences between standard (St) and chamber (Ch) environments.

(**b**) Lean body mass on day 7 of C_{12}TPP treatment. The values are the means ± S.E. of 6 mice in each group. The data were analyzed with Student's t-test.
